# Supplementary material for: Intrusion errors during verbal fluency task in amyotrophic lateral sclerosis
Source: PLoS One. 2020 May 29;15(5):e0233349. doi: 10.1371/journal.pone.0233349 (PMC7259757; doi:10.1371/journal.pone.0233349)
Supplement: Attachment — Submitted filename: Response to Reviewers.docx [file pone.0233349.s002.docx]

**Response to the editor and reviewers**

First of all, I would like to thank both reviewers for their helpful suggestions. I am sure that these changes will contribute to the improvement of the paper. The suggested revisions are described below mentioning point-by-point what changes have been made. After each suggestion, each answer is described written in bold.

*Reviewer#1*

*discussion, paragraph4: last sentence, "where the score in SVF and clusters decrease...moderate/severe alteration". It seem to be different with the results in Table 3.*

**Indeed, there was a mistake in the original manuscript and the text of paragraph 4 has been amended.**

*discussion, paragraph5: "As was previously, the patients without alterations in behaviour did not differ from the healthy population in the number of ords produced...", but the results didn't shown this content.*

**The analysis was performed but not included in the results section. Now it has been included and indeed, the Mann-Whitney *U* analysis reveals that there are not significant differences between those groups.**

*discussion, paragraph5:"we suggest that patients with moderate and severe alterations did not differ in the number of intrusion because...". From Table3 result, the severe group didn't produce enough word(PVF score -1.15), but moderate group, PVF score was similar with none or mild group, so how to explain the moderate group did not differ in the number of intrusion?*

**After discussing this particular we have decided to delete it due to lack of theoretical fundamentals of our explanations.**

*result, last paragraph: "ELA"--->"ALS"?*

**From native «Esclerosis Lateral Amiotrófica». The terms was replaced by ALS in the manuscript.**

*discussion, paragraph1:"ALS-FDT"--->"ALS-FTD"*

**The terms was replaced by ALS-FTD in the manuscript.**

*Reviewer#2*

*How were controls recruited?*

**Control group participants were recruited through public advisements in hospitals. This has been included in the original manuscript.**

*Exclusion/Inclusion: How were "critical levels of anxiety and/or depression at the time of the Evaluation" operationalized/assessed (questionnaire,* *cutoff ets)?*

**Critical levels of anxiety and/or depression were based on the HADS scores and its cutoff score stablished by the test ( ≥ 8 for the identification of suspicious cases and ≥ 11 for safe cases on both subscales of anxiety and depression). This particular has been included in the corrected manuscript.**

*Results:*

*It seems that you had 12 predictors in the stepwise model. Given that you only had 2x42 persons these are too many independent variables and the statistical test is probably underpowered.*

**We strongly agree with this point. Therefore, despite the clinical importance that it may have, this fact has been included as another limitation of the study.**

*What is the "ELA"group. This abbreviation was not introduced before.*

**From native «Esclerosis Lateral Amiotrófica». The terms was replaced by ALS in the manuscript.**

*Table 1. How was disease progression calculated?*

**The rate of disease progression was proposed by Kimura et al. (2006) and is calculated as follows: (48 – ALSFRS-R at time of diagnosis) / duration from onset to diagnosis (months)**

**This formula has been included in the original manuscript and the reference in the list.**

*Table 2. Using Mann Whitney implies that data are not normally distributed. Thus, you might consider to report median + IQR instead of mean + SD.*

**We fully agree with that statement and after the corrections, Tables are expressed in median and IQR.**

*Tables: please also Report the p-values for ns.*

**Non-significant P values are reflected in the new tables.**

*For Group comparisons please also Report the 95%CI*

**The 95%CI data is reflected in the new tables.**

*Did you observe any relationship between bulbar involvement (and consecutive higher disease progression and more frontal involvement) and dysexecutive components?*

**We had considered this fact from the beginning and after carrying out the analyzes regarding the type of disease onset (ie. bulbar vs. spinal) we have not found significant differences. However, due to its clinical importance question, we have included this in the Results and Discussion sections. Different studies have been cited and referenced in the manuscript.**

*Discussion:*

*A major limitation is the missing assessment of general cognitive function (eg ECAS, MoCa). Please comment on this.*

**We fully agree with the mistake of not using screening tests. In the corrected manuscript, their applications and virtues have been discussed in the Limitations section. Different studies have been cited and referenced in the manuscript.**
