# Supplementary material for: Intrusion errors during verbal fluency task in amyotrophic lateral sclerosis
Source: PLoS One. 2020 May 29;15(5):e0233349. doi: 10.1371/journal.pone.0233349 (PMC7259757; doi:10.1371/journal.pone.0233349)
Supplement: Attachment — Submitted filename: Response to Reviewers.docx [file pone.0233349.s003.docx]

**Response to Reviewers**

Dear Editors and Reviewers:

Thank you for your letter and for the reviewers’ comments concerning our manuscript. Those comments are very helpful for revising and improving our paper. We have studied comments carefully and have made correction which we hope meet with approval.

*Reviewer #1:*

*Three minor aspects remain:*

*It is more common to report ´median (IQR)´instead of ´IQR (median)´*
**Response:** We made the suggested changes in the tables.

*Now the authors state that their statistic is probable underpowered. I do not know if this is the right way. I am not a statistician, but I would prefer to use appropriate statistics for the sample size. I recommend to involve (statistical) editor or statistician to solve this issue.*

**Response:** After discussing this issue with more experienced colleagues, they are totally agree with the revisor’s caveat. For that reason, we choose only those variables which were statistically significant when comparing ALS group and the control group. This resulting in five variables which are more suitable for the analysis. Also, two subgroups were made after the discriminant analyisis and thier characteristics are described in the results section.

*I recommend to translate the raw data into English.*

**Raw data were translate into English.**

Once again, thank you very much for your comments and suggestions.

Prof. Manuel Perez
